# Synthesis, Structure, Properties, and Applications of Fluorinated Polyurethane

**DOI:** 10.3390/polym16070959

**Published:** 2024-04-01

**Authors:** Donghan Li, Lu Yu, Zhan Lu, Hailan Kang, Long Li, Shufa Zhao, Ning Shi, Shibo You

**Affiliations:** 1 College of Materials Science and Engineering, Shenyang University of Chemical Technology, Shenyang 110142, China; 2 Liaoning Provincial Key Laboratory of Rubber & Elastomer, Shenyang University of Chemical Technology, Shenyang 110142, China; 3 Shenyang Guide Rubber Products Co., Ltd., Shenyang 110141, China

**Keywords:** polyurethane, fluorinated polyurethane, high-performance materials

## Abstract

Fluorinated polyurethane (FPU) is a new kind of polyurethane (PU) material with great applicational potential, which is attributed to its high bond energy C-F bonds. Its unique low surface energy, excellent thermal stability, and chemical stability have attracted considerable research attention. FPU with targeted performance can be precisely synthesized through designing fluorochemicals as hard segments, soft segments, or additives and changes to the production process to satisfy the needs of coatings, clothing textiles, and the aerospace and biomedical industries for materials that are hydrophobic and that are resistant to weathering, heat, and flames and that have good biocompatibility. Here, the synthesis, structure, properties, and applications of FPU are comprehensively reviewed. The aims of this research are to shed light on the design scheme, synthesis method, structure, and properties of FPU synthesized from different kinds of fluorochemicals and their applications in different fields and the prospects for the future development of FPU.

## 1. Introduction

Polyurethane (PU), i.e., polycarbamate (-NHCOO-), is a block polymer composed of alternate soft (soft amorphous phase) and hard (hard crystalline phase) segments. PU is synthesized via the stepwise polymerization of polyhydroxy polymers (polyol) and polyisocyanates [1,2]. The soft segments are flexible chain segments, accounting for ~50%–90% of the total volume of the PU molecular chain. They are generally composed of polyester or polyether polyols and have a random curve shape at room temperature (20 °C). The hard segments are rigid chain segments, accounting for ~10%–50% of the total volume of the PU molecular chain. They are generally composed of polyisocyanates, small molecule diols (as the chain expander), and monohydric alcohols (as the capping agent). The high cohesion energy of aryl and urethane groups promotes intermolecular hydrogen bonding, which yields a rod shape of these hard segments at room temperature.

In addition, the thermodynamic incompatibility of the soft and hard segments in PU causes their dispersion and aggregation to form a microphase separation structure. This structure yields PU with unique mechanical and processing properties. Therefore, it is widely used in various products, such as coatings, adhesives, elastomers, foams, and leather [3].

PU was first synthesized by Otto Bayer [4] in the mid-1930s. Since then, it has been further developed into various types, including thermoplastic PU, flexible PU, rigid PU, and water-based PU. Thus, PU is one of the fastest developing and indispensable synthetic polymer materials. In recent years, with the development of science and technology, the weather resistance, corrosion resistance, degradability, and actual demand of PU under harsh and complex conditions have increased.

To mitigate these shortcomings, high-performance PU was synthesized by designing a molecular chain structure by introducing nanomaterials or chemical groups, such as polycarbonates and nitrogen-containing, phosphorus-containing, and fluorinated groups. Such PU materials are typically known as nanomaterial-based PU, polycarbonate PU, nitrogen-containing PU, phosphorus-containing PU, fluorinated PU (FPU), and polysiloxane PU [5,6,7].

Owing to its excellent chemical properties, fluorine has garnered considerable attention for the synthesis and application of high-performance polymer materials, particularly PU. The small atomic radius and high electronegativity of fluorine yields C-F bonds with a bond energy as high as 485.67 kJ/mol, which is considerably higher than that of C-C bonds, which is 332 kJ/mol [8,9]. The related constants of C, H, and halogen are shown in Table 1. When fluorinated chain segments are introduced into PU, they migrate to the PU-air interface and considerably reduce the surface-free energy of the material. The adjacent fluorine atoms are distributed in a spiral along the carbon chain, playing a good shielding role for the molecular chain. The as-obtained PU retains its mechanical properties and microphase separation structural characteristics and attains high hydrophobicity, oleophobicity, thermal stability, flame retardancy, and biocompatibility. This makes FPU stand out among other modified PU materials; therefore, it is widely used in coatings, clothing textiles, and the aerospace and biomedical fields [10,11]. FPU has garnered wide attention since it was successfully synthesized using diisocyanate and fluor diol by Lovelace [12] for the first time in 1958.

**Figure 1:**
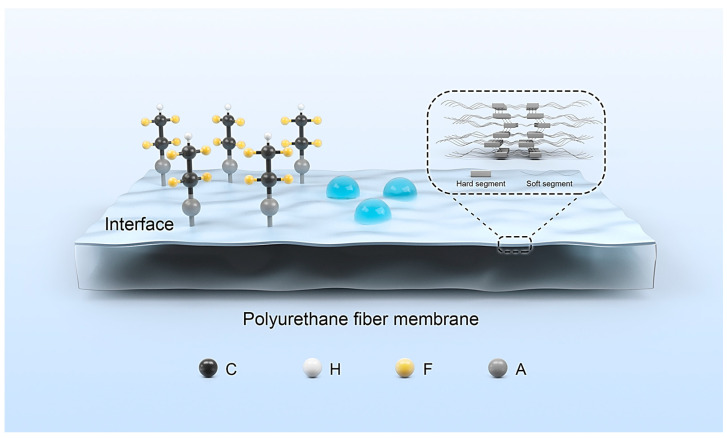
Schematic diagram of PU structure and migration of fluorinated chain segments.

To explore new materials that are resistant to liquid oxygen and noncombustible in pure oxygen, the Narmco Research & Department Division of the Whittaker Corporation (Simi Valley, CA, USA) developed several types of fluorinated isocyanates, fluorinated diols, polyesters, and polyethers in 1963, thereby expanding the applicability of FPU. To promote the development of the space industry led by the National Aeronautics and Space Administration, Minnesota Mining and Manufacturing, Dupont, the Ukrainian Institute of Polymer Science of the former Soviet Union and other organizations subsequently conducted more in-depth research on FPU. Furthermore, FPU has not only occupied a place in modified PU but has also become an independent research direction of fluoropolymers. (Figure 1 showed that schematic diagram of PU structure and migration of fluorinated chain segments).

**Figure 2:**
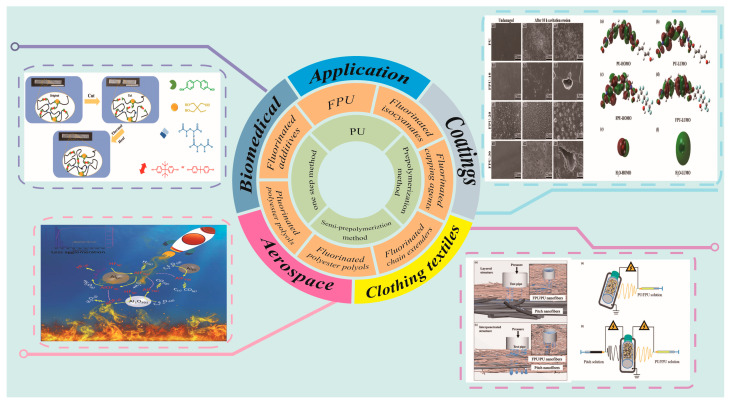
The synthesis method of PU and the introduction method of the fluorinated chain segment of FPU and its application. (Image of the self-healing process of the FPU spline: reprinted with permission from Ref. [13]. Copyright 2022, copyright Elsevier. Image of FPU coating and its model: reprinted with permission from Ref. [14]. Copyright 2022, copyright Elsevier. Image of the application of FPU in rocket launches [15]. Image of FPU/PU composite fabric permeation and its preparation method: reprinted with permission from Ref. [16]. Copyright 2019, copyright SAGE).

Although FPU has been extensively studied, its applications have not been comprehensively reported. Herein, the synthesis, structure, properties, and applications of FPU are comprehensively reviewed to highlight the design scheme, synthesis method, structure, and properties of FPU products and their applications in various fields. Moreover, the future development of FPU is prospected (Figure 2).

## 2. Synthesis of Typical PU

PU is traditionally synthesized using polyisocyanates and polyols containing two or more isocyanate groups [R-(N=C=O)_n≥2_] and a hydroxyl group [R-(OH)_n≥2_] via a polycondensation reaction. The synthesis involves a one-step method, a prepolymer method, and a semiprepolymer method. The one-step method involves the continuous processing of synthetic raw materials in proportion to a mixing reaction or chemical reaction process. The one-step method is simple, but the heat release is concentrated, and the resulting material strength is slightly lower than that obtained using the prepolymer method.

**Figure 3:**
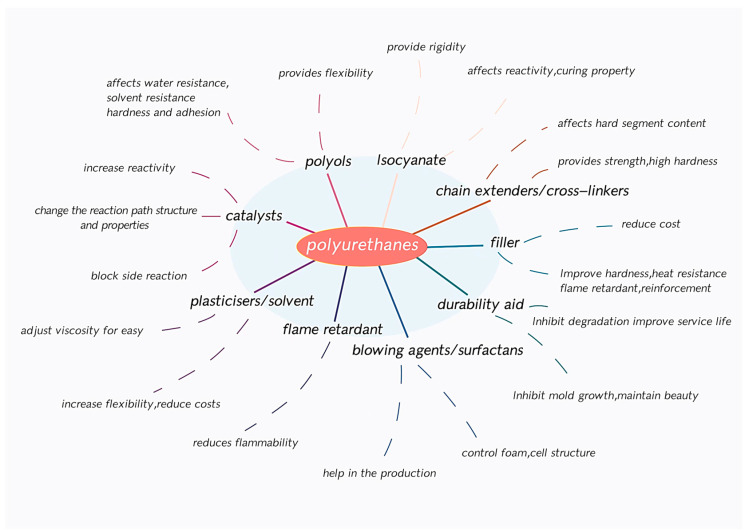
The components of PU and their roles.

In the prepolymer method, oligomer polyols and excessive amounts of polyisocyanate undergo a mixed reaction to produce PU prepolymers with low molecular weights and regular structures in the presence of chain extenders as the curing agent. The semiprecursor method was developed based on the aforementioned reaction, in which certain amounts of oligomer polyols and polyisocyanates are synthesized into a semiprepolymer. The remaining oligomer polyol is then mixed with the chain extenders, which then reacts with the semiprepolymer to form PU. Moreover, several methods, such as emulsification and self-emulsification (acetone, prepolymer dispersion, molten dispersion, ketoimine, and ketoazine), are used for synthesizing waterborne polyurethane (WPU). The properties of PU mainly depend on the types of polyisocyanates and polyols used for its synthesis. In this block polymer, the polyisocyanates and chain extenders form the hard segments, which yields the strength and hardness to the PU. The oligomer polyols, with a weak polarity, forms soft segments, which yield the elasticity and low-temperature properties of the PU. PU with different functions can be designed and synthesized simply by changing the content and synthetic raw materials used for synthesis, such as polyisocyanates, polyols, or additives. The components of PU and their roles are shown in Figure 3 [17,18,19].

### 2.1. Hard Segments

Hard segments usually affect the mechanical properties of PU, particularly its tensile strength, tear resistance, and hardness. Polyisocyanates and chain extenders are traditionally used as the main raw materials for synthesizing the hard segments in PU.

#### 2.1.1. Isocyanates

In 1849, the German chemist A. Wurzt prepared alkyl isocyanate via the metathesis of alkyl sulfate and potassium cyanate for the first time but did not find its suitable use, and in 1869, Gentier performed a preliminary determination of the isocyanate structure. In 1884, W. Hentschel produced isocyanates by reacting amines or (amine) salts with phosgene, which laid the foundation for industrial development. Isocyanates can be synthesized via Hoffman rearrangement, Curtius rearrangement, and Lossen rearrangement reactions [20,21].

The isocyanates used for PU synthesis are mainly aromatic, aliphatic, and alicyclic structures. Toluene-2,4-diisocyanate (TDI), diphenylmethane diisocyanate (MDI), hexamethylene diisocyanate (HDI), xylene diisocyanate (XDI), isophorone diisocyanate (IPDI), p-phenylene diisocyanate (PPDI), and tetramethyl-xylylene (TMXDI) are the most commonly used isocyanates; their structures are shown in Table 2. Aromatic isocyanates have a higher reactivity than that of aliphatic and alicyclic isocyanates. Due to the presence of a rigid aromatic ring, the hard segments have a higher cohesive energy but weak oxidation and UV resistance. These segments easily turn yellow and affect the aesthetics of materials. Thus, the types and content of polyisocyanates considerably impact the properties of PU [22,23,24,25].

Liu et al. [26] synthesized a series of PU materials with different hard segments from MDI, TDI, and HDI using the prepolymer method, and their microphase separation structures and properties were analyzed. The results show that the PU synthesized from flexible the aliphatic isocyanate, HDI, had a higher degree of phase separation, whereas that synthesized from the rigid isocyanate, TDI, had a lower degree of phase separation and better mechanical properties. However, excessive amounts of hard segments will affect the heat resistance of PU. Additionally, due to the strong micro-orientation ability of the PU synthesized using MDI and the high two-phase mixing degree, its tensile strength can reach up to a maximum of 26.8 MPa. The reactivity of the MDI-based PU is higher than that of the TDI-based PU, because the two -NCO groups in MDI are far apart, without any substituents nearby and are very active. If one of the -NCO group reacts, the other -NCO group still retains its high activity [27].

#### 2.1.2. Chain Extenders

Chain extenders (chain growth agents) are the most commonly used curing agents for PU synthesis and play an important role in the structure and morphology of the finished product. During PU synthesis, the chain extenders react vigorously with the -NCO group to form high concentrations of carbamate groups that form hard and stiff polymers. They also regulate the reaction rate of the system [28,29]. Furthermore, suitable chain extenders can improve the heat, chemical, and weather resistance of PU. They are mainly categorized as diols and diamines. Diols such as 1,4-butanediol (BDO), ethylene glycol (EG), diethylene glycol (DEG), and hydroquinone bis(2-hydroxyethyl) ether (HQEE) have lower molecular weights than PU prepolymers and a higher reactivity with the -NCO group. Carboxylated diols with hydrophilic groups can be used to synthesize WPU. 3,3′-dichloro-4,4′-diphenylmethane diamine (MO-CA) and diethyltoluenediamine (DETDA) are commonly used diamine chain extenders. They rapidly react with the -NCO group and are difficult to control; however, the as-synthesized PU has good mechanical properties. Notably, water is also a special chain extender. The hydrogen atom in the water molecule reacts with the -NCO group to form substituted urea, which is equivalent to a dual-functional chain extender. Thus, during PU synthesis, the moisture content of raw materials must be strictly controlled. However, when EG is used as the chain extender and if the hard segment content is considerably high, the as-derived biphenyl compounds are prone to degradation [27,30,31,32].

### 2.2. Soft Segments

Soft segments usually affect the mechanical properties and thermal properties of PU, particularly its elasticity and low-temperature performance. Polyols (oligomers) are generally used as an important raw material for the synthesis of PU soft segments.

#### Polyols

**Figure 4:**
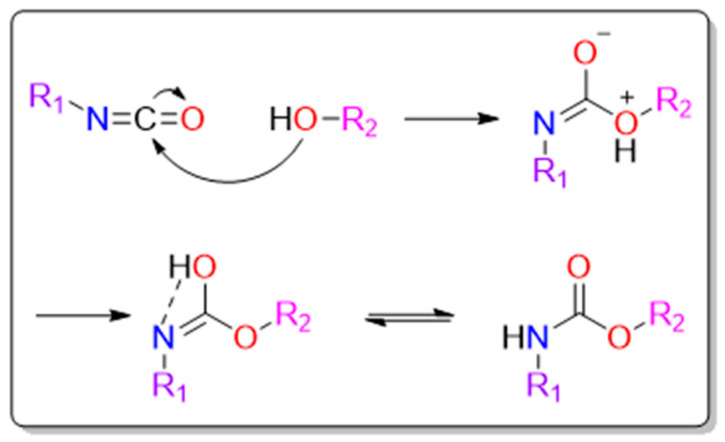
The reaction mechanism of isocyanates with alcohols.

Alcohols that contain two or more hydroxyl groups are called polyols. They can also contain functional groups such as esters, ethers, amides, acrylics, and metals [33]. Polyols usually act as soft segments and are used to synthesize PU components, which include polyether polyols; polyester polyols; and other oligomer polyols, such as polyolefin polyols, vegetable oil polyols (castor oil, castor oil-derived polyols, soybean oil polyols, palm oil polyols, and tall oil, etc.) [34,35,36], and rosin ester polyols, can also be used for synthesizing PU components. Their number-average molecular weight (Mn) is usually between 500 and 6000. Polyether polyols are generally prepared via the ring-opening homopolymerization or epoxy monomer copolymerization of the initiator (polyhydroxy, primary amine compounds or alcohol amines) with ethylene oxide (EO), propylene oxide (PO), butylene oxide (BO), etc., under the action of a catalyst [37,38]. Poly propylene glycol (PPG), poly-tetrahydrofuran glycol (PTMG), tetrahydrofuran propylene oxide copolymer glycol, and polyether polyols are commonly used polyether polyols. As the polyether-type PU has ether groups that rotate easily, compared with the polyester-type PU with relatively unstable ester groups in their molecular chain, they have a higher low-temperature flexibility and hydrolysis resistance. Polyester polyols are usually prepared via the condensation reaction of organic dicarboxylic acid (anhydrides or esters) and diols (including polyols). As their molecules contain a large number of ester groups, they have a stronger intermolecular bonding and higher cohesive energy (12.2 kJ/mol) than the ether groups (4.2 kJ/mol). The resulting soft segments also have strong intermolecular force, and the ester groups can form hydrogen bonds with the urea ester in the hard segments, promoting the mixing of the soft and hard segments. Polyester polyols have a stronger adhesion to polar substrates and higher viscosity than polyether polyols. The as-synthesized PU also have better mechanical properties. Polycaprolactone polyol, polycarbonate diol, conventional polyester polyol, and modified polyester are commonly used polyester polyols. The reaction mechanism of isocyanates with alcohols is shown in Figure 4. The oxygen atom in the alcohol acts as a nucleophile and attacks the carbon atom in the isocyanates, causing a nucleophilic-addition reaction. The resulting product undergoes an intramolecular proton transfer to form an imine acid intermediate, which then undergoes tautomerization to form a carbamate structure [27,39,40].

Although typical PU has a high mechanical strength, high elastic modulus, and high abrasion resistance, the enrichment of polar groups (hydrophilics) on its surface leads to poor weather resistance as well as humidity and heat resistance, which limit its applicational range. Therefore, new PU materials with enhanced properties have been synthesized via the route of a molecular chain structure design. Since this development, FPU has stood out. The introduction of fluorinated chain segments retained the original excellent performance and microphase separation structure characteristics of PU and endowed PU with excellent hydrophobicity, weather, heat, and flame resistance, and good biocompatibility. Therefore, FPU has currently become a research hotspot [41,42,43]. 

## 3. Design and Synthesis of FPU

Since 1958, when Lovelace [12] (UK) first synthesized and patented FPU from diisocyanate and fluorinated glycol, FPU have been studied extensively by researchers in various fields. Up to now, FPU with targeted performance can be synthesized precisely by designing fluorochemicals including fluorinated isocyanates, fluorinated capping agents, and fluorinated chain extenders as hard segments; fluorinated polyester polyols and fluorinated polyether polyols as soft segments; and fluorinated acrylate as additives. The methods of introducing fluorinated chain segments and their corresponding product characterization are shown in Table 3 [44,45,46].

### 3.1. Design of Fluorochemicals as Hard Segments

The fluorochemicals designed and synthesized for the hard segments of FPU mainly include fluorinated isocyanates, fluorinated capping agents, and fluorinated chain extenders [47].

#### 3.1.1. Fluorinated Isocyanates

Although there are industrial products of fluorinated isocyanates, their synthesis cost is high, the types are few, the preparation process is complicated, and the flexibility of the finished FPU product is poor, which leads to a great limitation in applications. 

**Figure 5:**
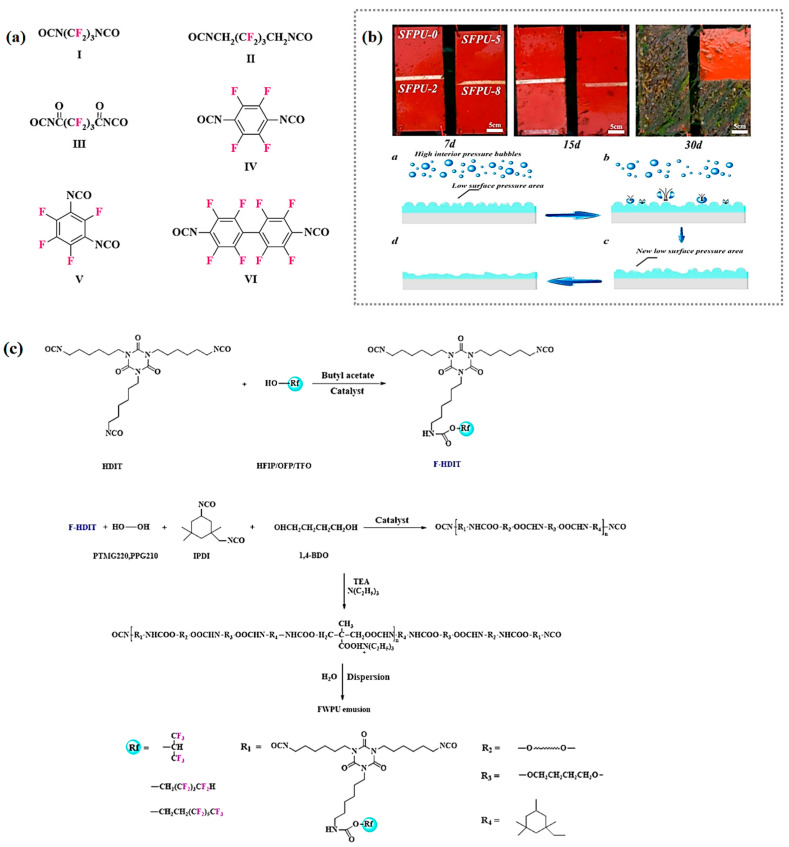
(**a**) Structure of six kinds of fluorinated diisocyanates [52]. (**b**) Digital photos of panels coated with FPU after static immersion in Yellow Sea for multiple days, and anti-cavitation mechanism diagram of FPU coating. (Reprinted with permission from Ref. [55]. Copyright 2021, copyright Elsevier). (**c**) Schematic diagram for the synthesis of WFPU [54].

In 1967, Hollander et al. [52], of Narmco R&D, first used hexafluoropentanediamine, hexafluoropentamethylene bischloro-formate, and tetrafluoro-phenylene bischloroformate, etc., as raw materials to separately synthesize six kinds of fluorinated diisocyanates, which included perfluorotrimethylene diisocyanate, hexafluoropentamethylene diisocyanate, and perfluoroglutaryl diisocyanate, etc., (Figure 5a). They were, respectively, used as fluorochemicals to synthesize FPU, but the results show that this kind of FPU has poor flexibility, low solubility, and only one type of the FPU could reach a thermal decomposition temperature of 300 °C (about 20 °C higher than typical PU), and the rest of the FPU showed a lower thermal stability than typical PU, which makes it difficult to be widely used in industrial production. To improve the problem of poor solubility, Lim [53] first used N-Ethyl-N-2-hydroxyethyl-per-fluorooctanesulfonamide and tris(6-isocyanatohexyl) isocyanurate to synthesize fluorine-modified diisocyanate. On this basis, anionic waterborne fluorinated polyurethane (WFPU) was synthesized by the direct dispersion method, and the results of this performance study show that the particle size of the WFPU increased from 174.3 nm to 239.7 nm with an increasing fluorine content. This may have been due to the fact that the increase in the amount of fluorinated isocyanate leads to stronger rigidity, and the increase in the viscosity of the liquid is not conducive to the fragmentation of the dispersed phase. The particle size of the WFPU decreased from 269.8 nm to 81.7 nm with an increasing neutralization degree. This was due to the increase in the number of hydrophilics in the WFPU with an increase in the neutralization degree.

For the FPU synthesized by the above method, its fluorinated chains are immobilized in the main chain of the macromolecule, which may lead to an undesirable surface enrichment of fluorine and a limited migration freedom. Additionally, the shorter fluorinated side chains (C_n_F_2n+1_ (n ≤ 3)) cannot be well-oriented, and the FPU performance improvements are further limited. In this context, Wen [54] first used 3,3,4,4,5,5,6,6,7,7,8,8,8-tridecafluoro-1-octanol and a hex-amethylene diisocyanate trimer to synthesize a fluorinated isocyanate trimer (Figure 5c). A series of WFPU with both flexible spacer layers and long fluorinated side chains were synthesized as hard segments by the prepolymerization method, and the effects of the fluorine content and fluorinated side chains on the properties of WFPU were investigated. The results show that with the growth in the length of the fluorinated side chains and the increase in the dosage of the home-made fluorinated isocyanate trimer, the hydrophobicity of the WFPU gradually increased and the water resistance gradually decreased. The contact angles of water and methylene iodide can be up to 121.8° and 90.7° respectively, and the lowest surface tension was 12.2 mN·m^−1^. The surface tension decreased, resulting in easy spreading. There was an increase of up to 20.9 °C in the initial decomposition temperature (temperature at 5% and 10% mass loss) compared with typical PU. The tensile strength decreased from 18.0 MPa to 5.8 MPa, and the elongation at break increased from 552.0% to 1220.1%. This may have been due to the plasticization of long fluorinated side chains, which disrupts the interaction between macromolecules. In general, by controlling the dosage of fluorinated isocyanates, it is possible to obtain an FPU with a low surface-free energy and great wetting ability. Figure 5b showed that a digital photograph of the FPU coating synthesised by Yang [55] with fluorinated isocyanate after many days of immersion in the Yellow Sea and the anti-cavitation mechanism of the coating. 

#### 3.1.2. Fluorinated Capping Agents

Fluorinated capping agents are primarily fluorinated monohydric alcohols with a low molecular weight and low surface energy. When introduced as hard segments into FPU, fluorinated capping agents rapidly migrate towards the polymer-air interface because of their terminal position. As a result, the fluorinated groups are anchored on the surface of the FPU, enhancing its surface properties while maintaining the original microphase structure of the PU. This allows for improved surface characteristics without compromising the material’s high mechanical strength [56]. However, there are reports [57] showing that its modified effect is not obvious due to the lower fluorine content, and until now, little has been reported on the effects of fluorinated capping agents on the performance of FPU.

**Figure 6:**
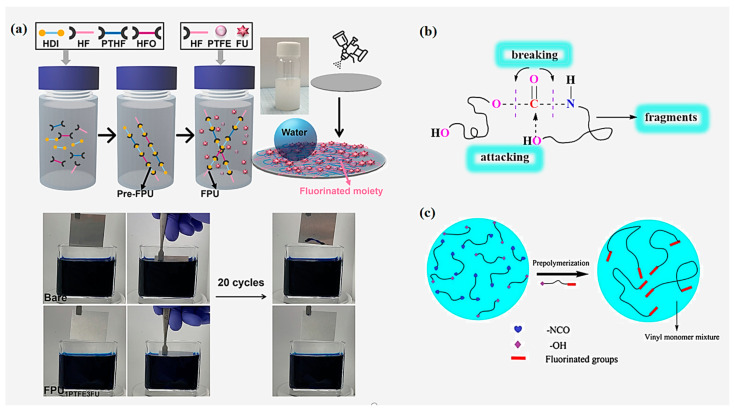
(**a**) Schematic of synthetic the FPU and the antifouling test by immersion in dyed water: bare substrate and FPU. (Reprinted with permission from Ref. [60]. Copyright 2024, copyright Elsevier). (**b**) Decomposition mechanism of PU [59]. (**c**) The position of fluorinated groups in FPU. (Reprinted with permission from Ref. [61]. Copyright 2014, copyright Springer).

Lahiouhl et al. [58] found that when the fluorinated chains are located in the side chain of the compound, its surface properties are significantly improved. Zhu [59] used monofunctional perfluorinated oligomers with fluorinated groups in both main and side chains (fluorinated monohydric alcohols FPOL, CF_3_CF_2_CF_2_O(CFCF_3_CF_2_O)_2_CFCF_3_CH_2_OH with a y-shaped structure), which were synthesized as capping agents with MDI and poly (ether glycol) (PEG) to form a telechelic FPU capped with perfluorinated polyether chains. The tests showed that unlike the main-chain FPU, perfluoropolyether-capped FPU have better flowability, and the fluorinated groups have a higher mobility. The hydrophobicity of the telechelic FPU significantly improved when the water contact angle increased from 85° to 113°, and its static water contact angle is comparable to that of pure polytetrafluoroethylene. Additionally, the perfluoropolyether chain segments can disrupt main-chain stacking and can the increase crystallization barrier; however, it will not affect the original microphase separation structure of PU. Under the same weight loss rate, the thermal decomposition temperature of FPU is about 15 °C higher than that of typical PU. This is due to the hydroxyl group at the end of the molecular chain of PU, which can break the urethane bond during the reaction process through inter- or intramolecular reactions, resulting in chain breakage(Figure 6b), and the introduction of fluorinated end-groups replaces the original end-hydroxyl groups, so that FPU shows better thermal stability.

Figure 6a showed that a schematic diagram of Kim’s [60] synthesis of a one-pot coatable FPU with a fluorinated capping agent and the antifouling test by immersion in dyed water: bare substrate and FPU; Figure 6c showed that the position of fluorinated groups in FPU synthesised by Wang [61] with fluorinated capping agents. Table 4 showed that the commonly used fluorinated capping agents for synthesizing FPU and their applications.

#### 3.1.3. Fluorinated Chain Extenders

The design and synthesis of FPU hard segments based on fluorochemicals are most commonly in the form of fluorinated chain extenders, and the main types primarily encompass small molecular fluorinated diols/fluorinated diamines. The FPU synthesized in this manner exhibits enhanced water resistance, flame retardancy, and thermal stability compared to unmodified PU [49].

**Figure 7:**
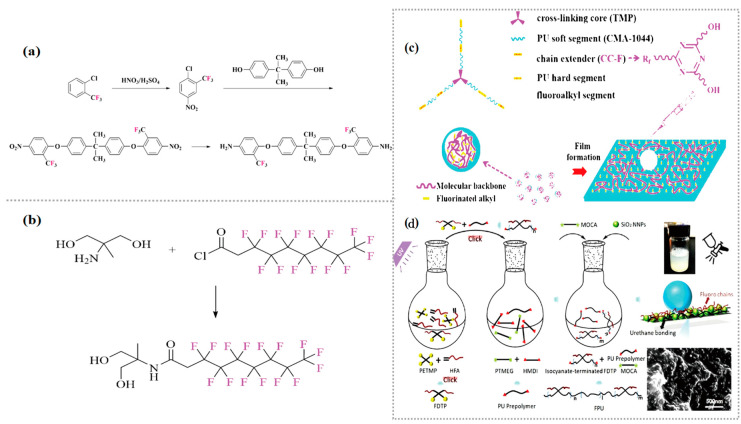
(**a**) Synthesis route of TF-type chain extender [64]. (**b**) Preparation of fluorinated chain extender with long side chains [65]. (**c**) Synthesis of novel triazine-based fluorinated chain extender-modified waterborne polyurethane hydrophobic films. (Reprinted with permission from Ref. [66]. Copyright 2021, copyright Elsevier). (**d**) Schematic illustration of fabricating SiO_2_ FPU superhydrophobic coatings. (Reprinted with permission from Ref. [67]. Copyright 2023, copyright WILEY).

Chen [63] used 2,2,3,3,4,4,5,5- octafluoro-1,6-hexanediol (OF) and 2,2,3,3-tetrafluoro-1,4-butanediol (TF) as chain extenders for polycondensation with HDI and poly-tetramethylene oxide (PTMO) to synthesize aliphatic FPU. By comparing this with FPU synthesized by BDO as the chain extender, the results show that elemental fluorine increases water resistance to some extent. The water contact angle of the OF type increased from 57.3 ± 2.2° to 59.7 ± 2.3° compared to the BDO type, and more fluorocarbon chains also significantly reduced the degree of platelet adhesion and platelet activation on the FPU surface. The synthesis of FPU using fluorinated diamines as chain extenders is scarcely reported in the relevant literature, and to fill the relevant theoretical gaps. Xu [64] used 2-chlorobenzotrifluoride and bisphenol A as raw materials and a home-made fluorinated diamine chain extender bis [4-(4-amino-2-trifluoromehyloxyphenyl)phenyl]propane (BAFPP) and used them to synthesize a series of FPU with different fluorine contents(Figure 7a). The results show that with an increase in the fluorine content, the water contact angle of the FPU increased from 67.3° to 86.2° and the water absorption decreased from 3.0% to 2.2%. The improvement in hydrophobicity and water resistance was attributed to the low surface-free energy of the fluorinated groups, which migrate and concentrate at the outermost interface of the polymer during synthesis. The peak heat release rate of the FPU synthesized on the basis of the chain extender TF was 282.9 W/g, which was much lower than the value of 537.2 W/g for ordinary PU, showing good flame retardancy. In terms of thermal stability, the thermal decomposition temperature of FPU is about 20 °C higher than that of PU at a 10% weight loss, which was attributed to the strongly polar -CF_3_ in the molecular chain, which requires more heat to break the polar bonding, and the strong polar bonding contributes to the microphase separation of the soft and hard segments of FPU, which has better thermal stability.

Xu [66] synthesised a novel triazine-based fluorinated diol CC-F.Subsequently, a series of WFPU (CC-FPUF-n) were synthesized using CC-F as the chain extender, and the synthetic schematic diagram is shown in Figure 7c. Figure 7d showed that a schematic illustration of fabricating SiO_2_ FPU superhydrophobic coatings summarised by Jiang et al. [67].

**Figure 8:**
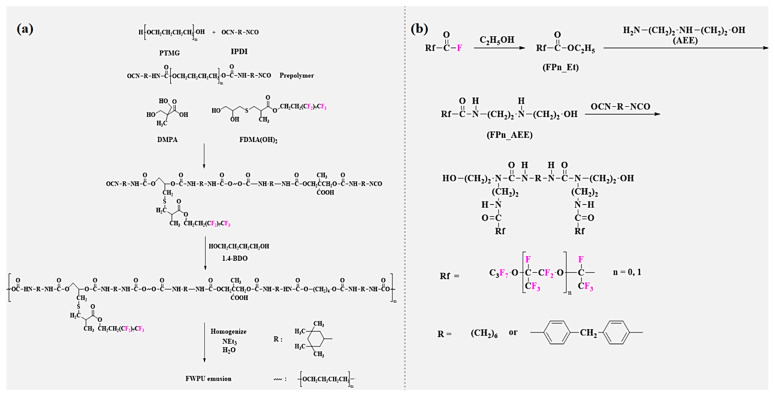
(**a**) Schematic of the synthesis of WFPU with fluorinated groups as side chains [68]. (**b**) Synthesis route of fluorinated gemini diol [69].

However, the fluorinated groups of the FPU synthesized in the above method exist in the main chain of the FPU. Although this can obviously improve the overall performance of the FPU, due to the restriction of the migration of the fluorinated groups, it is difficult to efficiently reduce the surface-free energy, and the performance of water and oil resistance will be affected. Owing to the excessive hydrophilic groups, the hydrophobicity of WPU is relatively poor, which limits its applicational scope. On this basis, Wu [68] synthesized a novel fluorinated chain extender (Figure 8a), which was dihydroxy-capped and which could introduce fluorinated groups as side chains into the WFPU by reacting 3-mercapto-1,2-propanediol (TPG) and 1,1,2,2-tetrahydroperfluorodecyl methacrylate (FDMA) in a simple and effective thiol-alkene click chemistry. Dedicated -NCO-terminated WFPU prepolymer tests have found that the water contact angle of WFPU increases from 67° to 104° with an increasing fluorine content, significantly improving the hydrophobicity of it, which is attributed to the migration of the fluorine groups to the interface, reducing the surface-free energy; however, due to the low dissociation energy of the introduced C-S bond, the thermal stability of WFPU is slightly decreased. In response to unsatisfactory thermal stabilization, Li et al. [65] applied a simple synthesis method to combine pentadecafluoro-octanoyl chloride and 2-Amino-2-methyl-1,3-propanediol into a novel long-segment side-chain fluorinated chain extender (AMPF) (Figure 7b). Subsequently, an environmentally friendly waterproof and breathable FPU membrane was synthesized by the prepolymerization method. The results of structural and performance studies show that the introduction of fluorine promotes the microphase separation of the soft and hard segments of FPU, and the tensile strength increases by approximately 28 MPa. As the fluorine content increases, the FPU membrane changes from hydrophilic (77.8 ± 2.7°) to hydrophobic (95.2 ± 2.1°), and the heat resistance improves by about 5% compared to ordinary PU.

Additionally, extending the length of the fluorinated group of the chain extenders is to advantageous for the thermal stability of FPU; a longer length of the fluorinated group would enhance the incompatibility of soft segments with hard segments, which would thus contribute to the microphase separation of FPU by hard-segment aggregation, thus requiring more heat to destroy the strong polar bonding.

Shi et al. [69] used home-made N-[2-[(2-hydroxyethyl) amino]ethyl]-perfluoroalkyl ether carboxylamide (Fpn-AEE) together with BDO as chain extenders (Figure 8b), and a series of novel environmentally friendly FPU were synthesized by the prepolymerization method. The surface and thermal properties were studied. The results show that the water contact angle of the FPU increased from 81° to more than 120° with an increase in the fluorinated dibasic diol ratio. The contact angle of tetradecane increased rapidly, and an excellent hydrophobic effect could be obtained. In addition, the introduction of fluorinated dibasic diols can lower the glass transition temperature of PU, and the microphase separation of the soft and hard segments of the PU is more obvious. Within a certain range, the decomposition temperatures of the T_smax_ and T_hmax_ of the soft segments and hard segments of FPU can reach 422 °C and 353.5 °C, respectively, which are higher than those of typical PU (33.7 °C and 26.5 °C), and this can significantly improve the thermal stability of FPU. However, the rigid groups may hinder the movement of hard segments, and the accumulation of hard segments is low, resulting in the lower thermal stability of MDI-type FPU than those of the HDI-type, and this study contributes to the development of FPU for practical applications.

### 3.2. Design Fluorochemicals as Soft Segments

The fluorochemicals designed and synthesized for the soft segments of FPU mainly encompass semi-fluorinated polyester polyols, semi-fluorinated polyether polyols, perfluorinated polyether polyols, or conventional polyether/polyester blends via the above fluorochemicals [70,71,72].

#### 3.2.1. Fluorinated Polyester Polyols

**Figure 9:**
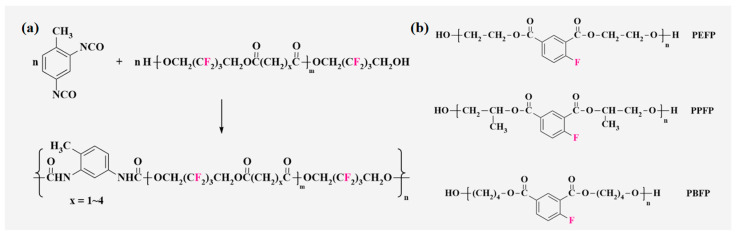
(**a**) Reaction of poly (hexafluoro-penta-methylene adipate) and poly (hexafluoropenta-methylene malonate) with 2,4-TDI. (**b**) Structural comparison of polyester- type FPU prepolymers [50].

Fluorinated polyester polyols are easy to hydrolyze under acidic and alkaline conditions due to the high surface energy of the ester group -COO-, so it does not improve the overall performance of PU, and the hydrophobicity and oleophobicity are much lower than polyether-type PU. In addition, owing to the high viscosity of the polyester polyols, their miscibility with other components during the synthesis process is poor, resulting in greater synthetic difficulties, so there are few reports on it. Polyester diols are generally prepared from small molecules of fluorinated diols with non-fluorinated dichloride and are then reacted with diisocyanate to synthesized FPU. It is important to note that when the halogen content in FPU is too high, it may be difficult to cure due to the high spatial potential resistance. Reaction of poly (hexafluoro-penta-methylene adipate) and poly (hexafluoropenta-methylene malonate) with 2,4-TDI is shown in Figure 9a.

Tamareselvy [50] used home-made 3-fluorophthalic anhydride, 1,2-propanediol (BDH) and BDO to prepare fluorinated polyester glycol (Figure 9b), and a series of polyester-type FPU were synthesized by reacting them with TDI and HDI, respectively. Their thermal stability, alkali resistance, hydrolytic stability, and hot melt adhesion with chlorinated and non-halogenated analogues were compared. The results show that all types of FPU have an increased onset decomposition temperature of more than 50 °C compared to typical PU, and tetra-halide PU has a better thermal stability than that of mono-halide PU. The hydrolytic stability of FPU is far superior to that of ordinary PU, which hydrolyzed to varying degrees only after 10 days in a 10% NaOH solution, but is poor relative to chlorinated PU. In addition, FPU shows the highest adhesion, and its peel strength can reach 1.68 × 10^6^ N·m^−2^. Therefore, FPU has a certain potential for application in related fields. 

#### 3.2.2. Fluorinated Polyether Polyols

Due to the low cohesive energy of the ether bond in the structure and being easy to rotate, polyether polyols can reduce the viscosity of the system and are more easily miscible with other components. This product has good low-temperature flexibility and hydrolysis resistance, compared with polyester-type PU that contain unstable ester groups, and it has better thermal stability and is commonly used in the synthesis of high-performance PU coatings [51].

**Figure 10:**
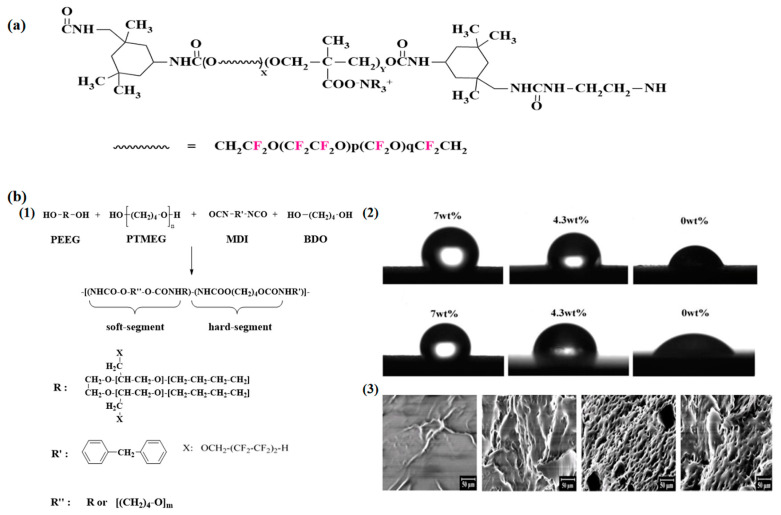

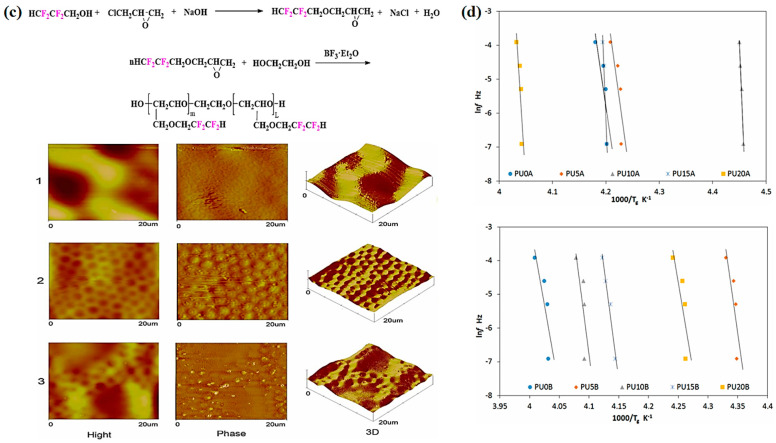
(**a**) Chemical structure of anionic polymer−repeating units of WFPU [75]. (**b**) (1) Schematic of the synthesis of polyether−type FPU; (2) Contact angle photographs of FPU elastomers with different fluorine contents to water and glycerol; (3) SEM plot of its variation with fluorine content. (Reprinted with permission from Ref. [76]. Copyright 2015, copyright Springer). (**c**) The reaction formula of fluorinate polyether diol and the AFM images of the microphase separation of FPU (observation domain: 20 mm). (1) PFGE/PBA (100/0); (2) PFGE/PBA (70/30); (3) PFGE/PBA (50/50). (Reprinted with permission from Ref. [7]. Copyright 2020, copyright Elsevier). (**d**) Simulation of the glass transition temperature of polyether−type FPU [77].

In the early stages, fluorinated polyether polyols were mainly prepared from 1,2 epoxy fluorinated compounds, as in, for example, the anionic- and cationic-initiated polymerization of 3,3,3-trifluoro-1,2-epoxypropane and its reaction with isocyanates to synthesize polyether FPU [73]. After Ausimont [74] first synthesized perfluoropolyether (PFPE) glycols (industry-named FomblinZ-DOL(HOCH_2_CF_2_O(CF_2_CF_2_O)_p_(CF_2_O)_q_CF_2_CH_2_OH) and FomblinZ-DOLTX(H(OCH_2_CH_2_)nOCH_2_CF_2_O(C_2_F_4_O)_p_(CF_2_O)_q_CF_2_CH_2_O(CH_2_CH_2_O)_n_H)), PFPE has been widely studied for its low surface energy, glass transition temperature, excellent thermal stability, and ability to enhance the multi-phase structure of cross-linked polymers, which significantly improve the service life of PU. Turri [75] used a ZDOL-type perfluoropolyether glycol (M_n_ = 1000–2000), bis (hydroxymethyl)propionic acid (DMAP), and IPDI as raw materials, and an anionic monomeric block-type WFPU (Figure 10a) was synthesized by the prepolymerization method. The results of this study show that most perfluoropolyether dispersions are stable for more than 12 months. Although perfluoropolyether itself is soft, high modulus membranes can be obtained due to the efficient phase separation of PU, and the lower the molecular weight, the higher the elastic modulus. Furthermore, WFPU basically shows a fully fluorinated surface, which provides the possibility of introducing a large number of fluorinated chain segments into WPU to improve the performance at a later stage. The process of synthesis of FPU from fluorinated polyether by Jia [76] and its water contact angle and SEM schematic are shown in Figure 10b.

AFM analyses of the microphase separation structure of polyether-type FPU (Figure 10c) were performed by Liu [7] and Król [77] modelled the glass transition temperatures of polyether-type FPU (Figure 10d).

Because most fluorinated coatings with excellent properties contain long perfluoroalkyl chains (C_n_F_2n+1,_ n = 6–10), there are problems, such as bioaccumulation and toxicity in nature, which have limited their use in the international community, and there is an urgent need to find alternatives. Based on this, Zhu [78] used the ring-opening polymerization of epoxy-butane to synthesize polyether diols containing CF_3_CF_2_CH_2_- and (CF_3_)_2_CH-. Two kinds of WFPU containing novel fluorinated short alkyl chains were synthesized by a reaction with IPDI. The results of this study show that the water contact angles of the two different FPU on the treated fabrics were 132° and 146°, which were almost superhydrophobic. The FPU containing linear CF_3_CF_2_CH_2_- showed better hydrophobicity and was promising as a new raw material to replace the long perfluoroalkyl side chain in the synthesis of fluorinated coatings.

To improve the comprehensive performance of FPU, the molecular chain of its soft segments can be designed to have both ether and ester bonds, and this new type of FPU can combine the advantages of polyester-type PU and polyether-type PU. Liu [7] first self-synthesized a fluorinated polyether diol (PFEG) to improve the mechanical properties. Polybutylene adipate (PBA) and PFEG were used together as soft segments, and a series of thermoplastic FPU were synthesized by the prepolymerization method, and the effects of the mass ratio of the soft segments and mass fraction of the hard segments on the mechanical properties of FPU were also investigated. The results show that as the ratio of the PFEG/PBA decreased, the elongation at break of the FPU increased from 89.1% to 634.3%, and the tensile strength increased from 6.61 MPa to 14.33 MPa. However, as the content of the hard segments increased, the tensile strength tended to increase and then decrease, and as the fluorine content increased, the heat loss peak of the FPU shifted towards higher temperatures.

### 3.3. Fluorinated Additives

With the iteration of FPU synthesis technology, which is different from traditional synthesis methods, the design and synthesis of FPU based on fluorinated additives mainly applies fluorinated acrylate to WFPU. The performance of acrylate has good complementarity with PU. Typical WPU often contains more hydrophilic groups, resulting in a higher surface energy. Fluorinated acrylate can significantly enhance the water and oil resistance, anti-adhesion, and other characteristics of PU [79,80,81]. In addition, the method of synthesizing FPU has practical flexibility because of the wide variety of fluorinated acrylates that can be synthesized relatively easily. The methods for synthesizing FPU from fluorinated acrylates can be classified into non-copolymerization and copolymerization methods. Among these, the non-polymerization method first synthesizes PU precursors containing hydrophilic groups and then uses an initiator to swell the fluorinated acrylate containing hydrophobic chain segments from the outside of the PU to the inside, initiating the polymerization reaction; however, in most products, PU and fluorinated acrylates exist independently of each other, and there is large core-shell dispersion and poor emulsion stability in WFPU. The copolymerization method can fully compensate for the shortcomings of the non-polymerization method, so that the core-shell cross linked structure of WFPU, which gives full play to the advantages of the monomer, limits the ability of chain segments to move, and the tensile strength and thermal properties of the material are further improved, and the film formation is more uniform. Therefore, it is the most widely used method to synthesize FPU from fluorinated acrylates [82,83,84].

**Figure 11:**
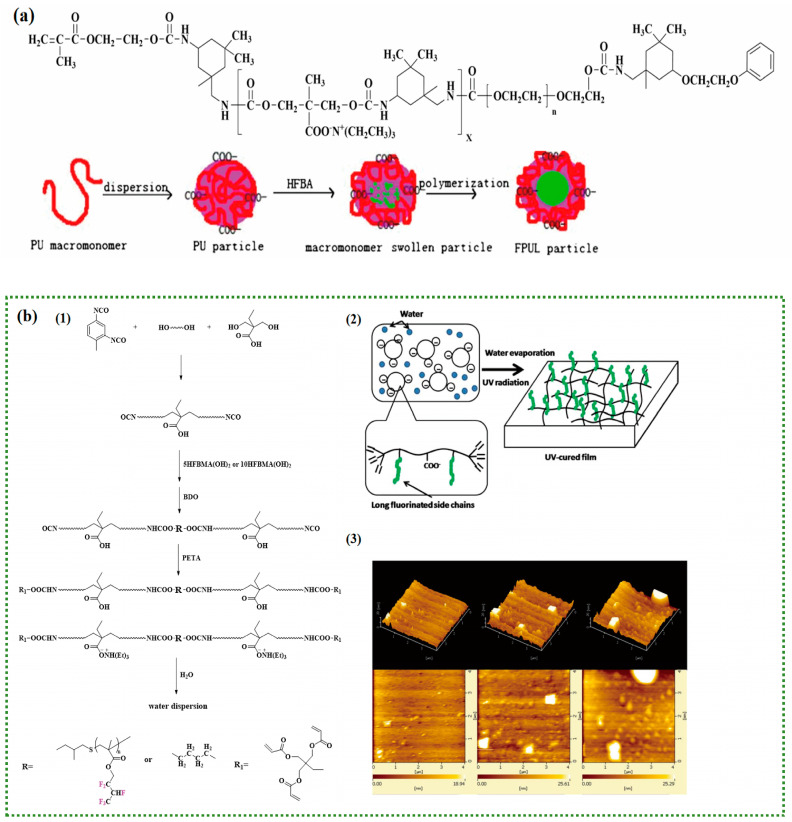

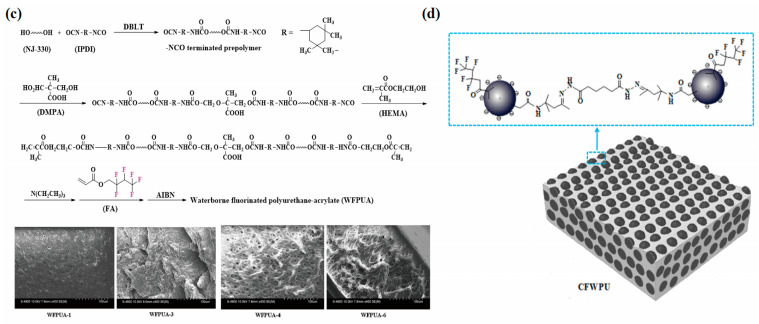
(**a**) Structure of the PU macromonomer and schematic illustration of the formation of novel FPU particle. (Reprinted with permission from Ref. [85]. Copyright 2007, copyright Springer). (**b**) (1) Synthesis of UV−curable WPU and WFPU; (2) schematic representation of UV−cured film formation and the migration of long fluorinated side chains; (3) AFM images of UV−curable WFPU. (Reprinted with permission from Ref. [87]. Copyright 2016, copyright WILEY). (**c**) The synthetic route of WFPU and the SEM micrographs of WFPU films. (Reprinted with permission from Ref. [88]. Copyright 2013, copyright Elsevier). (**d**) Structure of WFPU and crosslinked WFPU (CFWPU). (Reprinted with permission from Ref. [89]. Copyright 2016, copyright Elsevier).

Ionized double-bond capped PU macromolecules were first introduced as emulsifiers by Jiang [85] and then were self-emulsified by the copolymerisation method. It was used with hexafluoro-butyl acrylate to synthesize WFPU (Figure 11a). The results of this study show that the fluorine content of WFPU synthesized by self-emulsifying copolymerization has little effect on the particle size and surface charge and is suitable for the synthesis of a WFPU with a high fluorine content. The relatively regular microphase distribution appeared on the surface of the WFPU, and the contact angle test showed that the surface tension of the WFPU decreased significantly. However, with the deepening of research, conventional copolymerization methods cannot effectively control the composition of the polymer and chain length, etc. This has a greater limitation on the performance of the finished product. Based on this, Jiang et al. [86] improved the synthesis method by using self-made tetraphenyl ethanediol as a macromolecular initiator and were the first to use the iniferter method. A series of novel FPU, for biomedical applications, that were synthesized by the living radical polymerization of PU and hexafluoro-butyl acrylate were developed. Moreover, the fluorine content in the FPU could be easily regulated by modulating the hexafluoro-butyl acrylate content. The surface properties, thermal properties, mechanical properties, and oxidative stability of the new FPU were investigated. The results show that the monomer conversion of each component of the FPU was mostly dependent on the temperature, and nearly a 89% conversion was accomplished at 80 °C for 18 h. As the content of the hexafluoro-butyl acrylate increased, the water contact angle of the FPU reached up to 120° ± 2°, and the hydrophobic significantly improved. The glass transition temperature increased, presumably owing to the hydrogen bonding between the PU and hexafluoro-butyl acrylate. All FPU that in the test had good mechanical properties, with a maximum elongation at break of 1002.5% ± 125.6%. The FPU in an oxidizing environment did not show significant surface degradation compared to the drastic degradation of typical PU.

Tan et al. [87] prepared hydroxy acrylates with different chain lengths by polymerizing hexafluoro-butyl methacrylate with the chain transfer agent 1-thioglycerol radical and successfully synthesized UV-curable WFPU with long fluorinated side chains (Figure 11b). The results show that with the incorporation of fluorinated monomers, the roughness increased up to 3.99 nm. The surface energy of the WFPU decreased from 44.44 mN/m to 29.09 mN/m with only a small amount of fluorine, and the hydrophobicity of the WFPU significantly enhanced. The T_max_ increased from 312.1 °C to 322.0 °C, and the thermal stability enhanced. The adhesion between the UV-curable film and the substrate did not decrease with an increase in the fluorine content, and the adhesion on the surface of the polycarbonate, polyethylene terephthalate, and polymethyl methacrylate could reach 5 B. In addition, the synthesis process of this WFPU raw material is simple, and the synthesis and study of UV-curable WFPU with long fluorinated side chains can fill the theoretical gap of related properties. Wang [88] synthesised a series of WFPU from polyester polyol (NJ-330) and hexafluorobutyl acrylate (FA), the synthetic routes and SEM images are shown in Figure 11c; Zhong et al. [89] synthesised a novel crosslinked coating matrix with fluorinated acrylates, and fluorinated water-dispersed PU particles were uniformly distributed on the surface and in the interior, the structure of which is shown in Figure 11d.

## 4. Advances in the Application of Functionalized FPU

FPU has become the main developmental direction of functionalized PU, and it has been so for 60 years, ever since Lovelace [12] first synthesized FPU in 1958. Due to the introduction of fluorinated chain segments, FPU has excellent properties that cannot be compared with typical PU, such as its good thermal stability, flexibility, chemical resistance, blood compatibility, and unique low interfacial-free energy. Therefore, it is widely used in the coating, clothing textile, aerospace, and biomedical industries [81,90]. FPU has become a research hotspot in recent years.

### 4.1. Coatings

With the development of the coating industry, the international market has an urgent demand for high-performance coatings with excellent water resistance, weather resistance, aging resistance, and other characteristics. Furthermore, FPU coatings have excellent weather resistance, corrosion resistance, and chemical resistance. Its performance is much higher than ordinary synthetic resin coatings, and its service life is generally up to 20 years or more. Compared with other coatings, it is more resistant to sea spray corrosion, and its internal structure is more stable; therefore, it has a wide range of applications in the field of construction, aerospace, automobiles, and ships [91,92].

FPU coatings, which are mainly used in the construction field to protect buildings and their internal materials from rain and snow and other weather corrosion damage and can be constructed in the wet grass-roots level and form a waterproof layer, are non-toxic, tasteless, and non-harmful to human health [93]. Wang [94] synthesized a series of FPU for waterproof coatings for building materials using polyether diol (Mn = 2000) and TDI as raw materials and investigated the fracture morphology, hydrophobicity, and adhesion of the coatings. The results of this study show that the synthesized FPU coating systems are homogeneous, and the contact angle in deionized water can reach up to 94.5° due to the migration of fluorinated chain segments, showing excellent hydrophobicity and a good adhesion of the coating to the substrate. This kind of FPU can be well applied to waterproof coatings for building materials.

**Figure 12:**
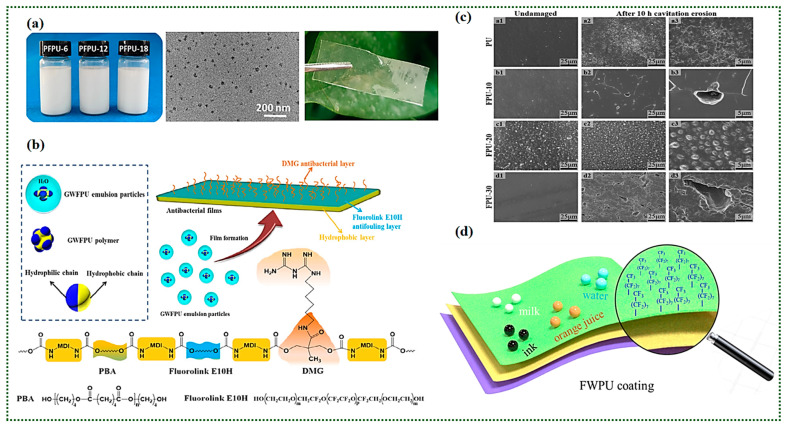
(**a**) FPU dispersion emulsion and finished film appearance. (Reprinted with permission from Ref. [96]. Copyright 2022, copyright Elsevier). (**b**) The schematic of antibacterial and antifouling WFPU films containing DMG and the corresponding chemical structure. (Reprinted with permission from Ref. [97]. Copyright 2018, copyright WILEY). (**c**) SEM images of the surface of the PU and the FPU coatings before and after exposure to cavitation erosion for 10 h (a1,b1,c1,d1 are undamaged PU/FPU coatings; a2,b2,c2,d2 are the PU/FPU coatings which exposure to cavitation erosion under 25 μm; a3,b3,c3,d3 are the PU/FPU coatings which exposure to cavitation erosion under 5 μm). (Reprinted with permission from Ref. [14]. Copyright 2023, copyright Elsevier). (**d**) Schematic image of the surface of WFPU coatings. (Reprinted with permission from Ref. [69]. Copyright 2021, copyright Elsevier).

In the field of marine and aerospace, in addition to the traditional protective and decorative functions of coatings, it is more important to have excellent resistance to high temperature, corrosion, water, oil, and weathering. Park et al. [95] synthesized a UV-curable FPU coating that can be used for the antifouling of ships using 4,4′-dicyclohexymethane-diisocynate, PTMG, and perfluoroalkyl acrylate 3,3,4,4,5,5,6,6,6,7,7,7,8,8,9,9,10,10,10-heptadecafluorodecyl methacrylate as the raw materials, and the surface properties and antifouling ability of FPU coatings were explored. The results show that the surface tension of the coating decreased from 23.4 mN/m to 14.2 mN/m due to the introduction of fluorinated chain segments. The water contact angle and diiodomethane contact angle of the coatings increased by 19.6° and 17.5°, respectively, which significantly improved the water and oil resistance of the PU coatings. This was attributed to its low surface tension, and after 78 days of immersion, the surface of the FPU coating had a weak adhesion of algae and barnacles, which are easy to remove, reflecting the excellent antifouling property of this coating. Figure 12a below showed that the FPU dispersion emulsion and finished film appearance; The schematic of antibacterial and antifouling WFPU films containing DMG and the corresponding chemical structure are shown in Figure 12b. Li [7] synthesised a series of FPU that with exceptional cavitation erosion resistance via hydroxy-terminated liquid fluorine elastomer, and the cavitation resistance of FPU was explored as a coating by SEM (Figure 12c); Figure 12d showed that the distribution of fluorinated chain segments on the surface of WFPU coating.

Chen [98] first prepared a novel fluorinated diol and poly(L-lactide) (PLLA) by Michael addition and a ring-opening polymerization reaction and synthesized a novel degradable FPU from it. The results of this study show that with an increase in the fluorine content, the water contact angle of the FPU increased from 71.12° to 108.24°, indicating a transition from hydrophilic to hydrophobic materials. The onset of the thermal decomposition temperature increased from 155 °C to 178 °C, and the hydrolysis ability of the FPU increased significantly with an increase in the PLLA content, which is an environmentally friendly biodegradable coating that can be used for ships.

Tang et al. [99] synthesized a ZnO-graphene oxide/WFPU nanocoating by mixing hydrophobically treated graphene oxide nanocomplexes with a homemade WFPU. The results show that this composite coating has excellent double hydrophobic properties, sea spray corrosion resistance, and friction resistance and can be well applied in the marine field. In addition, FPU can be used as aircraft skin paint in aircraft cabins and pipeline coatings, which is attributed to its good adhesion to alloy materials and good weather resistance and flexibility.

### 4.2. Clothing Textiles

**Figure 13:**
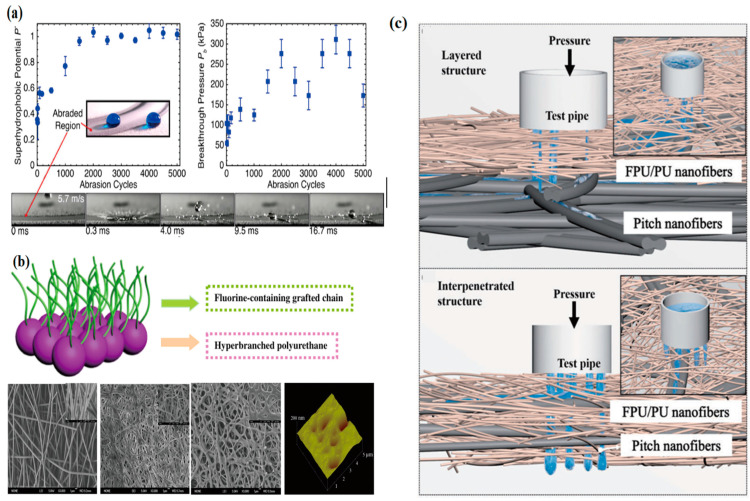
(**a**) The function of the number of abrasion cycles for the FPU/FPOSS coating after self-healing. The breakthrough pressure of the FPU/FPOSS coating as a function of abrasion, after self-healing. (Reprinted with permission from Ref. [103]. Copyright 2017, copyright ACS). (**b**) Fluorine-enriching membrane surface; SEM and three-dimensional AFM images of fluorination of hyperbranched polyurethane nanofibrous membranes. (Reprinted with permission from Ref. [104]. Copyright 2014, copyright Elsevier). (**c**) Image of FPU/PU composite fabric permeation. (Reprinted with permission from Ref. [16]. Copyright 2019, copyright SAGE).

To meet the increasing demand for textile functionality, fluorinated finishing agents are widely used in the textile industry. However, although fluorinated finishing agents prepared by traditional methods have good surface properties, they are harmful to human health due to the difficulty of the natural degradation of long fluorinated alkyl side chains, such as perfluoro-octane sulfonyl and perfluoro-octane compounds, and are subject to many limitations in their application. The use of FPU as a textile finishing agent can not only provide textiles with excellent hydrophobic, oleophobic, and anti-adhesive properties to meet the functional needs of textiles but also does not contain long fluorocarbon chain segments, which can effectively improve the impact of fluorinated finishes on humans, animals, and plants [100,101]. Zhu [102] prepared a series of fluorinated polyether diols based on the ring-opening polymerization of epoxy butane and polymerized them with IPDI to synthesize short-fluorinated carbon chain WFPU with different polyether diol matrices, and they were, respectively, applied to textile surface modifications. The results of this study show that with pentafluoro-propyl as the short fluorine chain, the water contact angle was as high as 146°, which was almost superhydrophobic, the elastic recovery performance enhanced by about 27%, and the tensile strength significantly improved. In the following figure, Figure 13a is the function of the number of abrasion cycles and the breakthrough pressure of the FPU/FPOSS coating after self-healing; Figure 13b is the SEM and 3D AFM images of fluorine-rich membrane surface and hyperbranched FPU nanofiber membrane; Figure 13c showed that the penetration image of FPU/PU composite fabrics.

In addition, the global market demand for waterproof and breathable functional fabrics is gradually increasing, and waterproof and breathable fabrics are widely used in the military, medical, and other fields due to their good breathability and ability to adapt to a variety of extremely harsh environments. Traditional waterproof and breathable fabrics only have excellent waterproof and windproof performance but have poor moisture permeability. The combination of FPU and electrostatic spinning technology can maintain the original performance of the fabric while obtaining excellent moisture permeability, providing the wearer good comfort [105,106].

Ge et al. [107] introduced perfluoro-1-decanol (CF_3_(CF_2_)_7_CH_2_CH_2_OH) (TEOH-8) to synthesize a novel FPU. The low surface energy perfluoro-alkane side chains were located at the end of the polymer, and the fluorinated chains efficiently migrated to the fiber surface. The microporous PU/FPU composite fiber membranes with strong waterproof and air permeability properties were prepared by electrostatic spinning technology. The results of this study show that, due to the fluorine element, the degree of adhesion between the spun filaments was reduced, the diameters were enlarged, the water contact angle reached 156°, and the oil contact angle reached 145°, and a strong hydrophobicity and oleophobicity were demonstrated. A air transmission rate of 8.46 L/(m^2^·s) and a water vapor transmission rate of 0.384 kg/(m^2^·h) were present due to the presence of micropores. This FPU showed good air permeability, and this kind of FPU can have a broader applicational prospect in the field of moisture-permeable fabrics.

### 4.3. Aerospace Industry

**Figure 14:**
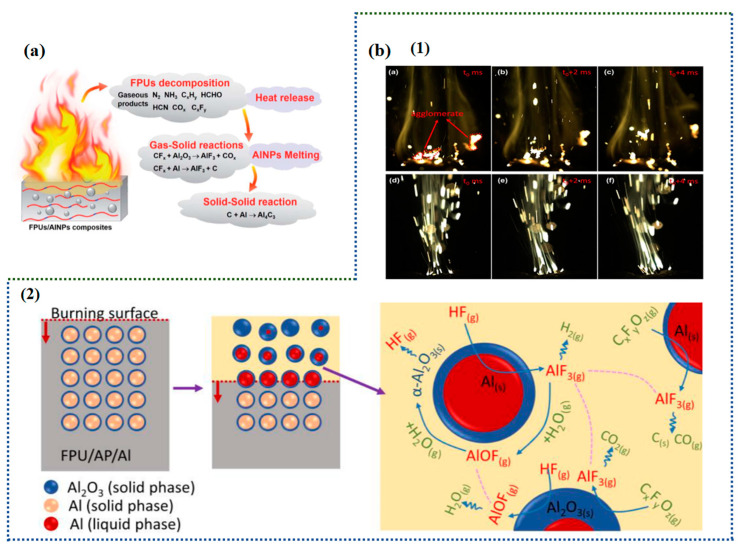
(**a**) A schematic illustration of the combustion reaction route of FPU/aluminum nanoparticle composites. (Reprinted with permission from Ref. [113]. Copyright 2022, copyright RSC). (**b**) (1) Images of the burning surfaces of different propellants at atmospheric pressure: (a–c) HPU/Al/AP; (d–f) FPU/Al/AP; (2) Schematic of the corresponding mechanism of FPU suppressing agglomerate [15].

Propellant technology for aerospace vehicles is one of the indicators of a country’s advanced space technology, and it plays a vital role in the stability of engine operation and the safety of the aircraft operations. For launch vehicles and spacecrafts that use liquid hydrogen and liquid oxygen as their propellant, since liquid hydrogen and liquid oxygen propellants are highly susceptible to evaporation and escape, leading to serious accidents and huge losses due to fuel leakage, it is necessary to select a sealing material that has both liquid-oxygen compatibility and excellent physical properties. FPU has good chemical inertness and self-extinguishing and liquid oxygen compatibilities, which is to the introduction of the fluorine element, with a good overall performance and can be used as an excellent low temperature sealing material [108,109]. In addition, the use of fluorine or its derivatives as additives in various propellants, explosives, and pyrotechnics, etc., has been widely recognized [110]. To adapt to different engine operating environments, research on fuel velocity modifiers for rocket propellants is becoming a new trend. The results of this study show that binders with a high pyrolysis temperature can significantly increase the temperature of the burning surface and reduce the burning speed of the propellant. In contrast to traditional inorganic burn rate modifiers, FPU burn rate modifiers do not drastically reduce the amount of energy produced by the propellant and have a superior thermal stability compared to PU [111]. Xu et al. [112] synthesized a series of fluorosilicone WPU and tested their performance. The results show that the maximum heat release rate and total heat release of the WPU decreased significantly with an increase in the fluorinated monomer. The flame resistance of the material greatly improved, while the thermal stability and residual sputum rate of the WPU also significantly improved. In the figure below, Figure 14a is the schematic illustration of the combustion reaction route of FPU/aluminum nanoparticle composites; Figure 14b showed that the surface images of different propellants burning at atmospheric pressure and the mechanism of FPU inhibition of agglomeration.

In addition, how to improve the combustion efficiency of the Al powder is also a hot topic of research at present, as Al powder is the most commonly used metal fuel in solid rocket propellants, but pure Al powder has a low melting point and tends to liquefy before ignition, which prolongs the ignition period. Owing to the viscous force of the propellant burning surface, Al powder and other components of the propellant are prone to agglomeration, fusion, and coalescence. This can seriously affect the propulsion efficiency. In severe cases, this can lead to an accumulation of hot particles on the inner walls of the engine and nozzle, ultimately causing flight failure [114,115]. FPU can be used to coat the Al powder, as its decomposition of small molecules of fluorinated compounds can be fluorinated with Al to produce AlF_3_, and the per unit of heat release is 80% more than Al_2_O_3_ [116].

Zhang [117] synthesized nano-aluminum/fluorinated PU composites based on the azide-alkyne click reaction. The results of this study show that Al or Al_2_O_3_ and the FPU decomposition of small molecules of fluorinated compounds at a 370 °C reaction, the formation of AlF_3_, the temperature of which overlaps with the reaction temperature of aluminum thermite, can reduce the ignition point of the Al powder, at 20 wt% (nano-aluminum in the FPU). The total exothermic amount reaches 2082 J/g, and the sublimation temperature of AlF_3_ is 1277 °C, which is much lower than the boiling point of Al_2_O_3_ 3000 °C, and it can effectively reduce the formation of the solid products of Al_2_O_3_.

### 4.4. Biomedical Industry

**Figure 15:**
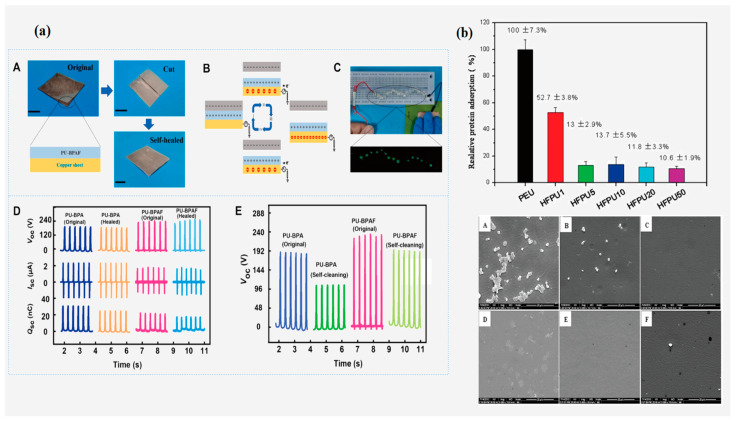
(**a**) (A) Photograph and schematic diagram of self-healable triboelectric nanogenerator (SH-TENG) (FPU). (B) Schematic diagram of SH-TENG working mechanism. (C) Demonstration of SH-TENG based on FPU driving LED lights. (D) Electrical output performance of the SH-TENG based on FPU and PU before and after self-healing. (E) Self-cleaning performance of TENG based on FPU and PU. (Reprinted with permission from Ref. [13]. Copyright 2022, copyright Elsevier). (**b**) Relative human fibrinogen adsorption on phosphorylcholine FPU (P-HFPC) and poly (ether urethane) (PEU) blend surfaces determined from enzyme-linked immunosorbent assay with ordinary PEU as a reference (mean 6 ± SD%); SEM pictures of P-HFPC and PEU blend film surfaces after contact with PRP for 1 h. (A), PEU; (B), HFPU1; (C), HFPU5; (D), HFPU10; (E), HFPU20; (F), HFPU50. (Reprinted with permission from Ref. [122]. Copyright 2011, copyright WILEY).

As an outstanding representative of stretchable elastomers, PU shows great potential for applications in wearable electronics [118]. It is widely used in various medical devices due to its good physical and chemical stability, mechanical properties, and biocompatibility. However, medical PU often causes a variety of adverse biological reactions, such as thrombosis, coagulation, protein adsorption, etc., in the human body. Usually, the surface modification of PU is used to reduce the formation of thrombus and related inflammations, and how to further improve the antithrombotic and human adaptability of PU has become a hot spot of research [119,120]. The introduction of fluorinated compounds into medical PU can significantly improve the oil resistance of the material, will not absorb lipids in the body and low surface properties, so that the fluorine atoms are enriched in the surface of the material to achieve the purpose of preventing the degradation of oxygen media on the body of the material, which can be used in the field of biomedicine [121]. In the following figure, Figure 15a shows a demonstration of the relevant test tool for self-healing/self-cleaning FPU and its self-healing/self-cleaning performance graphs; Figure 15b shows the relative human fibrinogen adsorption data on the FPU surface and SEM image of the membrane surface.

Jia [13] synthesized a multifunctionalized FPU from bisphenol AF (BPAF), PTMG, MDI, and trimethylolpropane, which is a new material with efficient self-healing properties and mechanical strength that can be used in wearable electronics. The results show that the introduction of the C-F bond increases the energy of the covalent bond and raises T_d5%_ from 253 °C to 260 °C, and both the FPU and ordinary PU show elastic behavior at 100% strain. In terms of self-healing performance, the self-healing efficiency of the FPU improved by 5% and 14% after 1 h at 80 °C and 90 °C, respectively, compared with that of the normal PU, and by 5% at 100 °C, with a self-healing efficiency of as high as 100%. In addition, the FPU significantly increased the open-circuit voltage and self-cleaning capability of the SH-TENG (increasing the efficiency from 62% to 83%), and as a self-healing and stretchable conductor, it can also be used in flexible electronic devices.

Tan et al. [123]. blended FPU with ordinary PU and studied its blood compatibility to obtain a high fluorinated surface to further improve the biocompatibility and biostability. The results show that the migration of the fluorinated chain segments to the surface of the material was easier in the blends. The surface fluorine content of FPU10% was about 121 times the theoretical bulk fluorine content, the ratio of the surface fluorine content to the theoretical bulk fluorine content for FPU50% was 23, the water contact angle was more than 109.3°, and the CH_2_I_2_ contact angle was more than 92.5°, showing excellent water resistance. The blood compatibility of the blends was significantly higher than that of typical PU in a platelet adhesion test, showing good applicational prospects.

## 5. Conclusions and Outlook

As a widely used polymer material with a low surface energy, excellent mechanical properties, flexibility, and corrosion resistance and good biocompatibility, FPU has become the focus of PU modification research in recent years. FPU can be precisely synthesized by designing fluorochemicals including fluorinated isocyanates, fluorinated capping agents, and fluorinated chain extenders as hard segments; designing semi-fluorinated polyester polyols, semi-fluorinated polyether polyols, perfluorinated polyether polyols, or conventional polyether/polyester blends via the above fluorinated compounds as soft segments; designing fluorinated acrylates as additives, and changes to the production process to satisfy the needs of the coating, clothing textile, aerospace, and biomedical industries for materials that are resistant to hydrophobicity, weathering, heat, and flames and with good biocompatibility. Here, the synthesis, structure, properties, and applications of FPU are comprehensively reviewed, with the aim to shed light on the design scheme, synthesis method, structure, and properties of FPU synthesized from different kinds of fluorochemicals including their products and applications in different fields. Furthermore, FPU has not only occupied a place in modified PU but has also become an independent research direction of fluoropolymers.

Although FPU research has made some progress, it still faces challenges in practical applications. Firstly, the preparation process of fluorinated compounds for synthesizing FPU is complicated, and the synthesis conditions are harsh, and the price is high, which makes it difficult to realize large-scale industrialized applications. Secondly, there are still shortcomings in the performance of fluorinated compounds currently developed. To obtain FPU with excellent performance, it is necessary to accurately design the mechanism and structure of fluorinated compounds, which is a challenging process. In short, high-performance FPU will become a strong support for the development of new materials in the world, with huge performance advantages and broad prospects.

## Figures and Tables

**Table 1:** Related constants of C, H, and halogen [8,9].

	C	H	F	Cl	Br	I	At
Bond length (×10^−10^ m)	1.70	1.2	1.35	1.85	1.96	2.16	-
Electronegetivity	2.55	2.10	3.98	3.16	2.96	2.66	2.20
Number of valence electrons	2	1	5	5	5	5	5
Ionization energy (kJ·mol^−1^)	1086.5	1319.85	1689.83	1257	1139.9	1008.4	890 ± 40
Electron affinity (kJ·mol^−1^)	121.7	72.78	328.1	348.5	324.5	295.1	233
C-X bond length (×10^−10^ m)	1.53	1.091	1.32–1.43	1.72–1.85	1.87–1.96	2.13	-
C-X bond energy (kJ·mol^−1^)	332	416.90	485.67	326.80	276	240	-
Atomic polarizability (α, cm^3^/10^−24^)	1.76	0.66	0.68	2.58	3.05	4.7/5.35	-

**Table 2:** Commonly used isocyanate structures and their corresponding PU product features [22,23,24,25].

Code	Structure	-NCO Content%	Product Features
TDI	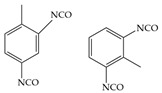	48.2	Good thermal stability
MDI	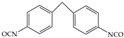	33.5	Good thermal stability Good moldability Good mechanical properties
HDI	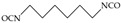	49.7–49.5	Good mechanical properties Good chemical resistance Good weather resistance Good adhesion
XDI	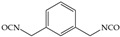	44	Good light stability
IPDI	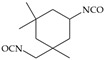	37.5–37.8	Good light resistance Good chemical resistance Good weather resistance Good abrasion resistance
PPDI	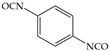	52.5	Good moisture resistance Good heat resistance Good oil and tear resistance
TMXDI	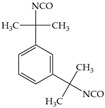	34.4	High strength High adhesion Good flexibility resistance Good yellowing resistance Good acid resistance Durable

**Table 3:** Methods of introducing fluorinated chain segments and their corresponding product characterization.

Fluorochemical	Characteristics	Ref.
Fluorinated isocyanates	Difficult to synthesize; high cost	[27,44]
Fluorinated capping agents	Low fluorine content; the modification effect is not obvious	[47]
Fluorinated chain extenders	Excellent water resistance, flame retardancy, and thermal stability	[48,49]
Fluorinated polyester polyols	High surface energy; unsatisfactory water resistance	[50]
Fluorinated polyether polyols	Excellent hydrophobicity; excellent hydrolytic stability	[51]
Fluorinated acrylate-non-copolymerization	Large core-shell dispersion in PU emulsions; poor emulsion stability	[46]
Fluorinated acrylate-copolymerization	Excellent water resistance and thermal stability; good mechanical properties	[46]

**Table 4:** Commonly used fluorinated capping agents for synthesizing FPU and their applications [62].

Fluorinated Capping Agents	Applications
F(CF_2_)_n_OH (n = 6~12)	Hydrophobic, oleophobic, and anti-adhesion fabric finishes
F(CF_2_CF_2_)_n_CH_2_CH_2_OH (n = 3~7)	Hydrophobic, oleophobic, and anti-adhesion fabric finishes Emulsion polymers
F(CF_2_CF_2_)_n_CH_2_CH_2_SH	Hydrophobic and oleophobic fabric finishes
C_7_F_15_CH_2_OH	Rigid insulating foam
HOCH_2_CF_2_CF_2_OCF(CF_3_) CF_2_OCF_2_=CF	Elastomer
C_6_F_13_(CH_2_)_2_S(CH_2_)_3_OH	Fiber cladding material
